# Laudatio for Distinguished Scholar Dr. Aaron T. Beck

**DOI:** 10.32872/cpe.6871

**Published:** 2021-06-18

**Authors:** Claudi L. H. Bockting

**Affiliations:** 1 Amsterdam UMC, University of Amsterdam, Amsterdam, The Netherlands

On behalf of the European Association of Clinical Psychology and Psychological Treatment (EACLIPT), I am honored to have the opportunity to award Dr. Aaron T. Beck (MD) with the European ‘*Diamond Distinguished Contributor to Psychological Intervention*s *Award’*.

On July 18, 2021, Dr. Beck will celebrate his 100th birthday. As a psychiatrist and scientist, he spent almost his entire career on reducing human suffering. With a medical and academic career spanning more than 70 years, 600 published articles, 25 books, and numerous awards, it is without doubt that Dr. Beck has greatly influenced and shaped our current thinking on psychopathology and clinical practice beyond the measurable. Albeit, measurability was of utmost concern to him during his career. Originally starting out as a neurologist after his medical training at Yale, particularly liking the precision of this field, he soon found himself becoming absorbed in psychoanalysis. Carrying over his interest in empirical work, he later widely explored and rigorously tested the psychoanalytic model of depression as a psychiatrist at the University of Pennsylvania. Other than expected he did not find evidence for the psychoanalytic concept, but rather unraveled the core assumptions of cognitive therapy. Due to his unremitting work, psychological interventions became more evidence-based, client-focused, and accessible to a wide variety of people with different conditions across the globe. Today, Cognitive Behavior Therapy (CBT) is the most studied psychotherapy (>2000 studies on CBT) for most mental health problems globally. Even more so, his Cognitive Behavioral theoretical model has led to groundbreaking insights on the etiology, maintenance, and recurrence of psychopathology.

Dr. Beck is not only the founding father of CBT, he also played a crucial role in demonstrating time and time again that research and clinical practice go hand in hand. That is, already in the early years of Dr. Beck, the concept scientist practitioner was ‘a given’. Most people who had the pleasure to see Dr. Beck on stage will recall his onstage role-plays. Without hesitation, until recent times he was always prepared to do a role-play in public at a conference or at events of the Beck Institute. In each role-play, Dr. Beck managed to give the audience the impression it was a piece of cake to conduct CBT. We all know better: It requires a lot of training. Nevertheless, it is indeed doable with the right amount of practice, which Dr. Beck was always aware of. One of the greatest achievements of Dr. Beck together with the Beck Institute, and probably one of the ingredients of the therapy’s success, is that CBT is highly transferable by training. The worldwide dissemination of CBT demonstrates clearly that CBT is highly transferable, even across different cultures.

**Figure:**
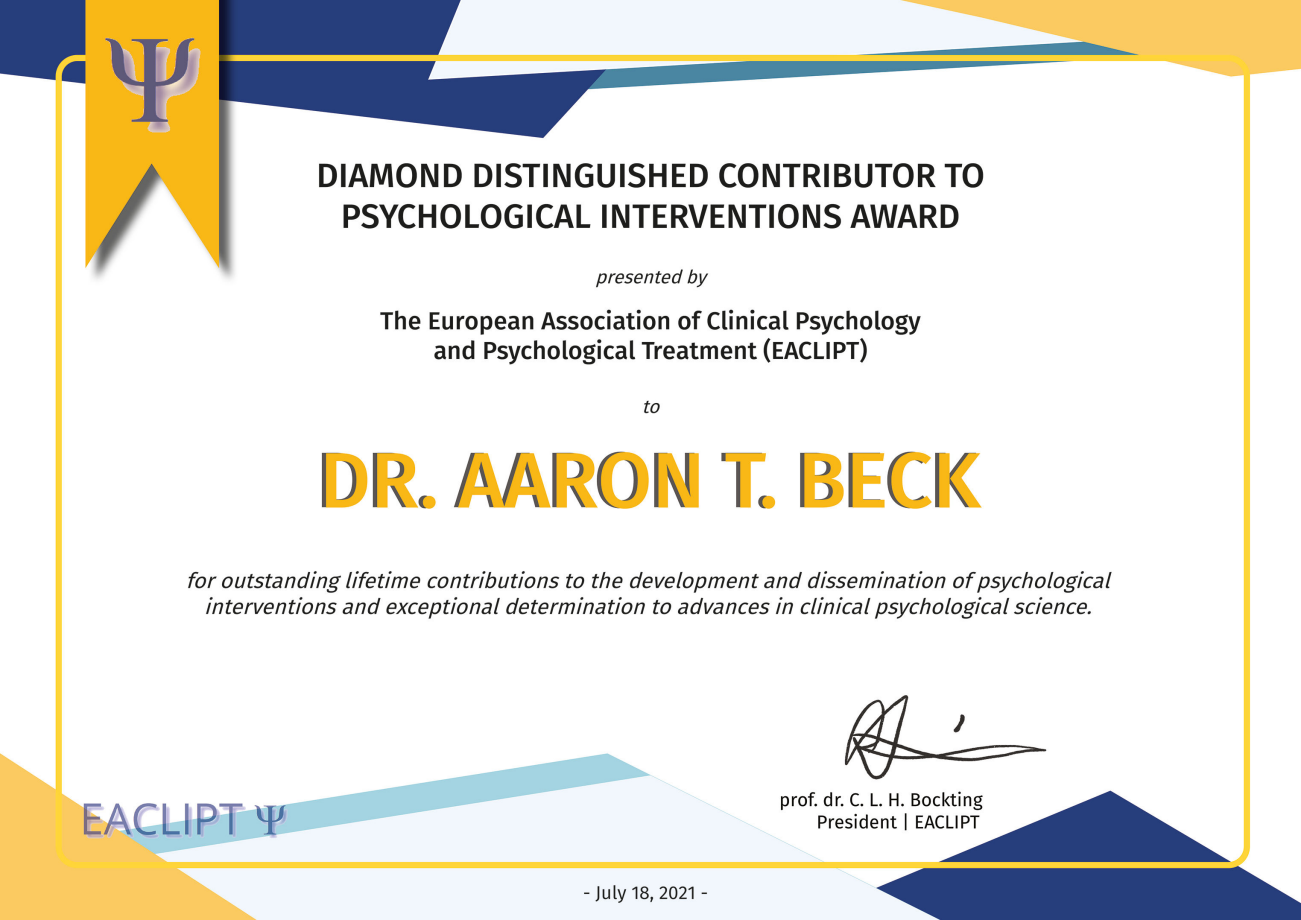
Dr. Aaron T. Beck Received the Diamond Distinguished Contributor Psychological Interventions Award by the EACLIPT

On a personal note, I vividly remember my first introduction to Dr. Beck when he received an honorary membership at the Dutch association for CBT. I was invited to join for dinner with Dr. Beck and a small group, and I was shocked to find out I was seated next to him. As a classical Dutch person (usually too direct), I had to actively inhibit my urge to immediately confront him with my slightly provocative questions that challenge the CBT model. Given all the research on inhibition, you will most likely know how hard it can be to inhibit these questions once they are on top of your mind. They can almost become intrusive, and even rebounce once you attempt to suppress them. So, well aware that pure suppression was not an option, I had to choose a more problem-focused approach. Therefore, I decided to discuss something completely different with Dr. Beck to distract my own mind. And what could be more impartial, universal and pleasing, than talking about music? Obviously, having my mental set of Beck associations activated, I couldn’t think of any other artist than the famous American musician Beck. So, my opening line was: “Do you know the very popular song Loser, in which Beck sings ‘*I am a loser baby (so why don’t you kill me)*’?”. We discussed that the song might indeed be inspired by CBT. Could it be that this song could even lead to cognitive restructuring in listeners? Taken together, it includes the identification of rigid negative beliefs, evaluating the evidence, as well as the formulation of alternative beliefs! After this small talk, of course, my suppressed thoughts rebounced – how could they not? Fortunately, Dr. Beck was happy to discuss all my burning questions, as he has always been after. For instance, why not intervene immediately on beliefs/schema level, instead of starting working on thought level before going there? He gave the only right answer: ‘That is an empirical question. You should study it’. This is, most certainly, another way in which Dr. Beck influenced science and clinicians: transfer curiosity to an empirical question and study it. I indeed did study this, later on in several trials. I can only imagine the large number of people he inspired throughout his life, and continues to do. Dr. Beck’s lifework is living and still developing.

By the way, Dr. Beck asked me to send him a disk of the song, and I did. He later told me that he didn’t know the song, but liked the idea of using music or other means to evaluate beliefs on a large scale. He was and is more than willing to provide feedback on articles and research. Hereby, he teaches us all an important lesson: curiosity should never stop. After all this time, he still serves as an inspiration to the scientific community, numerous scientist practitioners, clinicians all over the world. More importantly he contributed and still contributes significantly to reduce human suffering of many individuals all over the world that suffer from mental health problems and mental health conditions.

The EACLIPT is proud that Dr. Beck accepts our European ‘*Diamond Distinguished Contributor to Psychological Intervention*s *Award*’.



**Claudi Bockting**
*Professor of Clinical Psychology in Psychiatry*
*Amsterdam UMC/University of Amsterdam*
*President of EACLIPT*



